# Clozapine-induced myocarditis: a case report and literature review

**DOI:** 10.1192/j.eurpsy.2022.1891

**Published:** 2022-09-01

**Authors:** I. Angélico Ferreira, M.A. González Fé, M.D.M. Marqués Pastor, C. Roset, L. Torres

**Affiliations:** Hospital Universitario Son Espases, Psychiatry, Palma de Mallorca, Spain

**Keywords:** myocarditis, clozapine, clozapine-induced

## Abstract

**Introduction:**

We present the case of a male patient, 47 years old, diagnosed with schizophrenia, that was admitted at our hospital presenting a confusional state, with agitation, motor discoordination and difficulty breathing. At the blood analyses there was evidence of an increase in cardiac enzymes. The clinical manifestations had begun 5 days before, with slight leucocytosis showing in a routine blood test made after initiating clozapine, followed by fever, vomiting and progressive impairment of general clinical state.

**Objectives:**

To describe a case of clozapine-induced myocarditis, which is a known, but rare, side effect of clozapine and to do a brief review of the existing knowledge on this matter.

**Methods:**

The authors undertook an article review using PubMed database and a thorough analysis of the clinical case.

**Results:**

The hypothesis of clozapine-induced myocarditis was the main diagnosis considered since the beginning, nevertheless, a thorough clinical examination and complementary tests were made and all the previous psychopharmacological treatment was suspended. The final diagnosis was based on the clinical presentation (fever, vomiting, shortness of breath, confusion and impairment of general state), the elevation of CRP, PCT and TnI and findings on echocardiogram that suggested myocarditis (moderate systolic dysfunction of the left ventricle due to global hypokinesia and a non dilated left ventricle).

**Conclusions:**

The clinical manifestations observed, the results of the complementary diagnostic tests and the review of the existing literature, allowed to make the diagnosis of clozapine-induced myocarditis. We find of considerable importance to continue to publish and study this matter as it is still insufficiently known.

**Disclosure:**

No significant relationships.

